# Response to Wagner et al.: phosphodiesterase-2—anti-adrenergic friend or hypertrophic foe in heart disease?

**DOI:** 10.1007/s00210-016-1301-z

**Published:** 2016-09-27

**Authors:** Anna Zoccarato, Laura H. Fields, Manuela Zaccolo

**Affiliations:** 1 Department of Cardiology, Cardiovascular Division, British Heart Foundation Centre, King’s College London, London, UK; 2 Translational Medicine and Therapeutics, John Vane Science Centre, Charterhouse Square, Queen Mary University of London, London, UK; 3 Department of Physiology, Anatomy and Genetics, University of Oxford, Oxford, UK

In Zoccarato et al. (Zoccarato et al. 2015), we provide extensive evidence that inhibition of PDE2 counteracts cardiac myocyte hypertrophic growth. By measuring cell size, nuclear NFAT-translocation, protein synthesis, protein to DNA ratio and expression of hypertrophic markers, we demonstrate that pharmacological inhibition of PDE2, unlike inhibition of PDE3 or PDE4, inhibits the hypertrophy induced by norepinephrine (NE); and that overexpression of PDE2, but not overexpression of PDE4 or PDE3 isoforms, is sufficient to induce hypertrophic growth. We show that the effect of pharmacological inhibition of PDE2 is recapitulated by its genetic knock down (KD) using siRNA. We further demonstrate that the effect of PDE2 KD is specific as the phenotype can be rescued by overexpression of a siRNA-insensitive PDE2 construct. In addition, we find that overexpression of a catalytically inactive version of PDE2 also counteracts hypertrophy, an effect that is due to displacement of localised active endogenous PDE2. We confirm the anti-hypertrophic effect of PDE2 inhibition both in neonatal and adult ventricular myocytes and both in rat and mouse models. Crucially, we demonstrate in vivo that pharmacological inhibition of PDE2 counteracts hypertrophy in a mouse model of pressure overload and that in vivo overexpression of PDE2-mCherry, but not of its catalytically inactive mutant, induces cardiac hypertrophic growth in rat cardiac myocytes. Based on the above evidence, we suggest that inhibition of PDE2 may represent a novel therapeutic approach to control cardiac hypertrophy.

Indeed, our findings are difficult to reconcile with the observation, reported by Mehel et al., that overexpression of PDE2 in adult rat ventricular myocytes reduces the cellular hypertrophy induced by in vitro treatment with NE (Mehel et al. 2013). We suggested (Zoccarato et al. 2015) that this discrepancy may be only apparent and could be explained by the fact that in Mehel et al., the effect of PDE2 overexpression in NE-treated cells was presented as relative to the effect of overexpression of PDE2 in the absence of any additional stimulus. Such normalisation of the data prevents evaluation of the effect of PDE2 overexpression per se*.* In Wagner et al. (Wagner et al. 2016), the authors present the same data where now the effect of overexpression of PDE2 is shown relative to the effect of overexpression of GFP alone. They make the point that, in their hands, there is no evidence of a pro-hypertrophic effect of PDE2 overexpression; and propose that other factors, including differences in the constructs, in the transfected PDE2 isoform or in its cellular localisation may explain the discrepancy.

While there are indeed three different isoforms of PDE2 (PDE2A1, PDE2A2 and PDE2A3) for which a different subcellular localisation has been described (Lobo and Zaccolo 2016), this cannot account for the difference in the results that we are discussing here as in both studies the same isoform, PDE2A2, was overexpressed.

Other differences in the constructs and/or protocols, however, may be involved. Mehel et al. expressed PDE2A2 using a bicistronic adenovirus encoding EGFP (Ad-PDE2-GFP). This results in the expression of PDE2A2 and EGFP as separate entities, whereas, in Zoccarato et al., a single fusion protein (PDE2A2-RFP or PDE2A2-mCherry) was overexpressed. In addition, in Mehel et al., after isolation, the myocytes were infected for 24 h before application of NE for additional 24 h. In Zoccarato et al., the myocytes were exposed to the virus and simultaneously treated with NE for a total of 24 h in culture (Fields et al. 2015). As a result, a different level of transgene expression may be expected with the two protocols. This may be particularly relevant when considering that overexpression of GFP alone has been shown to induce hypertrophy in a dose-dependent manner (Huag et al., Nature Medicine, 2000), and the longer the myocytes are allowed in culture after infection, the higher the risk that this non-specific hypertrophic effect may occur. If overexpression of GFP induces per se myocyte hypertrophy, the additional effect of overexpression of PDE2 may be more difficult to detect. We noticed that in Fig. 2A, Wagner et al. expressed the effect of PDE2 overexpression as relative to overexpression of EGFP. It would be interesting to know how the size of cells overexpressing EGFP compares to control non-infected cells. In Zoccarato et al., we overexpressed a non-tagged versions of PDE2A2 (Zoccarato et al. 2015) and found a significant pro-hypertrophic effect compared to non-infected cells. Where a red fluorescent protein tagged PDE2 was overexpressed in Zoccarato et al., to mitigate the potential confounding effect of overexpression of the fluorophore we compared the effect of overexpressing PDE2A2 with the overexpression of its catalytically inactive mutant, two constructs, that except for two point mutations in the enzyme catalytic site, are otherwise identical and give similar levels of transgene expression (Stangherlin et al. 2011). We found that only the wild type, but not inactive enzyme, induced cardiac myocyte hypertrophy (Zoccarato et al. 2015).

**Fig. 1:**
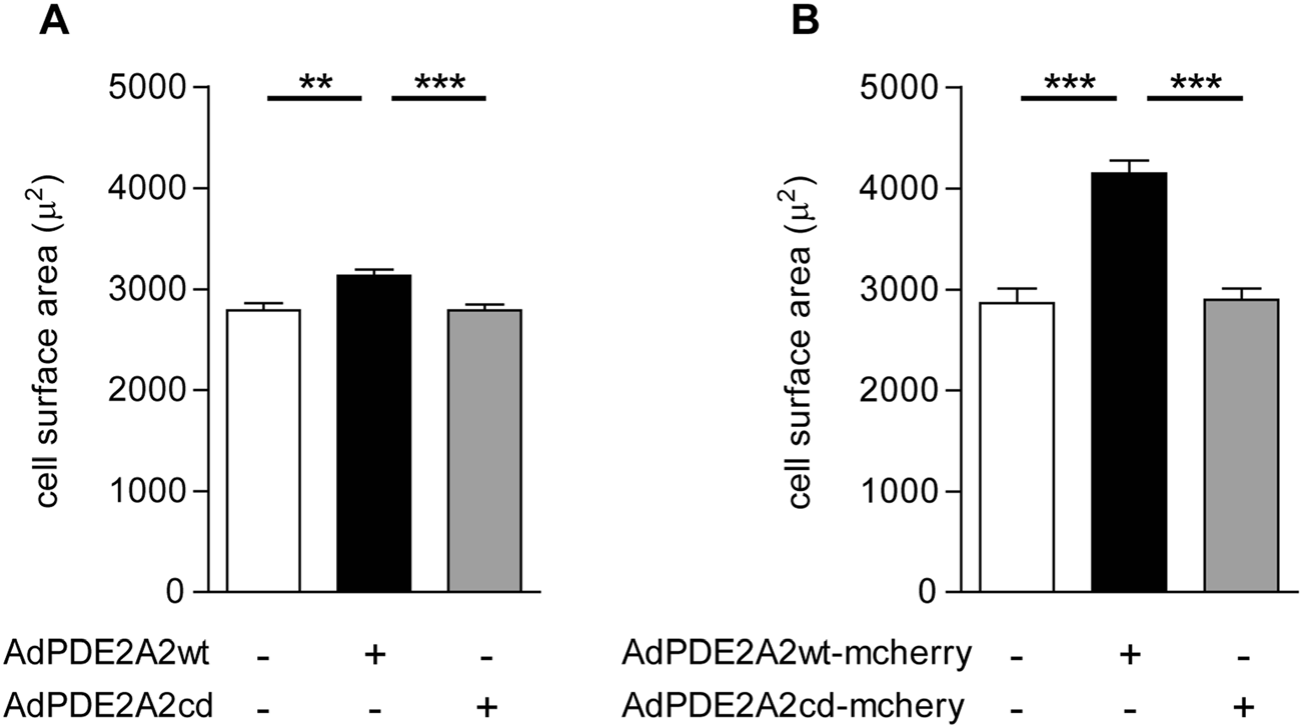
**a** Cell surface area measured for control adult rat ventricular myocytes (non-transduced) and myocytes transduced in vitro with AdV5/PDE2Awt or AdV5/PDE2A catalytically dead (Zoccarato et al. 2015). **b** Cell surface area measured for control myocytes and myocytes transduced with AdV5/PDE2Awt-mCherry or AdV5/PDE2A catalytically dead-mCherry. Data are expressed as mean ± s.e.m. and are the summary of three independent experiments (number of cells analysed ≥25). One-way ANOVA and Tukey’s multiple comparison tests were performed. **p ≤ 0.01; ***p ≤ 0.005

In our hands, both overexpression of mCherry-tagged and of un-tagged versions of PDE2A2 results in hypertrophy (Fig. 1). There is a smaller pro-hypertrophic effect on overexpression of un-tagged PDE2A2 compared to overexpression of the mCherry-tagged variant. However, one should keep in mind that the efficiency of viral infection is not 100 %, and the lack of a fluorescent marker makes it impossible to identify the cells that express the transgene from those that do not. As a consequence, a number of non-infected (and therefore non-hypertrophic) cells are included in the calculation of the average size in the experiment with un-tagged PDE2A2. In our system, it seems unlikely that expression of the fluorescent protein mCherry may induce hypertrophy per se, as cells expressing the catalytically inactive PDE2A2cd-mCherry are indistinguishable from control, non-infected cells.

In conclusion, our extensive data from both in vitro and in vivo experiments and results from both enhancement and inhibition of PDE2 activity, all concur in identifying PDE2A as a hypertrophic foe. Future work, including experiments using transgenic and KO mice, will certainly further contribute to clarify the role of PDE2 in cardiac pathophysiology.
